# Navicular and fifth metatarsal base stress fractures: An illustrated anatomical review

**DOI:** 10.1002/ksa.70413

**Published:** 2026-05-22

**Authors:** Xiuqi Liu, Choon Chiet Hong, Francisco Reina, Anna Carrera, Enric Alcolea‐Madera, Jorge Javier Del Vecchio, Miki Dalmau‐Pastor

**Affiliations:** ^1^ Department of Orthopedic Surgery Affiliated Hospital of Zunyi Medical University Zunyi Guizhou China; ^2^ Human Anatomy and Embryology Unit, Department of Pathology and Experimental Therapeutics School of Medicine and Health Sciences University of Barcelona Barcelona Spain; ^3^ Department of Orthopaedic Surgery National University Hospital Singapore Singapore; ^4^ Yong Loo Lin School of Medicine National University of Singapore Singapore Singapore; ^5^ Medical Sciences Department, Clinical Anatomy, Embryology and Neuroscience Research Group (NEOMA), Faculty of Medicine University of Girona Girona Spain; ^6^ Podiatry Unit School of Medicine and Health Sciences University of Barcelona Barcelona Spain; ^7^ MIFAS by GRECMIP: Minimally Invasive Foot and Ankle Society Merignac France; ^8^ iMove Traumatology Tres Torres Barcelona Spain; ^9^ Cirurgia Ortopèdica i Traumatologia (COT), Hospital d'Olot i Comarcal de la Garrotxa Girona Spain

**Keywords:** anatomy, fifth metatarsal, navicular, stress fractures

## Abstract

The navicular bone and the proximal fifth metatarsal (M5) play a vital role in maintaining the structural integrity and biomechanical stability of the foot's medial and lateral columns, respectively. Both bones' unique anatomical configuration, intricate mechanical environment and limited vascular supply make specific locations particularly vulnerable to stress fractures. Moreover, the combination of mechanical loading patterns and restricted perfusion in the navicular and proximal M5 further contributes to delayed healing, nonunion and refracture. Therefore, finding suitable treatment options for the different damage patterns involving both bones is critical for reducing complications and improving clinical results. This paper aims to clarify the anatomical and biomechanical features underlying these fractures to improve diagnosis, prevention, and clinical management in high‐risk populations.

**Level of Evidence:**

N/A.

**Abbreviations**
ICNL: inferior calcaneonavicular ligament
LB: lateral band
M5: fifth metatarsal
MPOL: medioplantar oblique ligament
PBT: peroneus brevis tendon
SLC: spring ligament complex
SMCNL: calcaneonavicular ligament
TPT: tibialis posterior tendon

## THE NAVICULAR BONE

Located in the medial aspect of the midfoot arch, the navicular bone is a crucial part of the tarsal bones. It forms the medial half of the midtarsal joint, while the calcaneocuboid joint forms the lateral half. It serves as a crucial anatomical bridge between the forefoot and hindfoot, playing an essential role in maintaining foot stability, transmitting mechanical loads, and supporting body weight during gait and stance [86]. Because of its complex anatomical connections, diverse articular surfaces, and comparatively limited blood supply, the navicular is particularly susceptible to a variety of pathological conditions in clinical practice, including stress fractures [28].

### Bony anatomy

**Figure 1:**
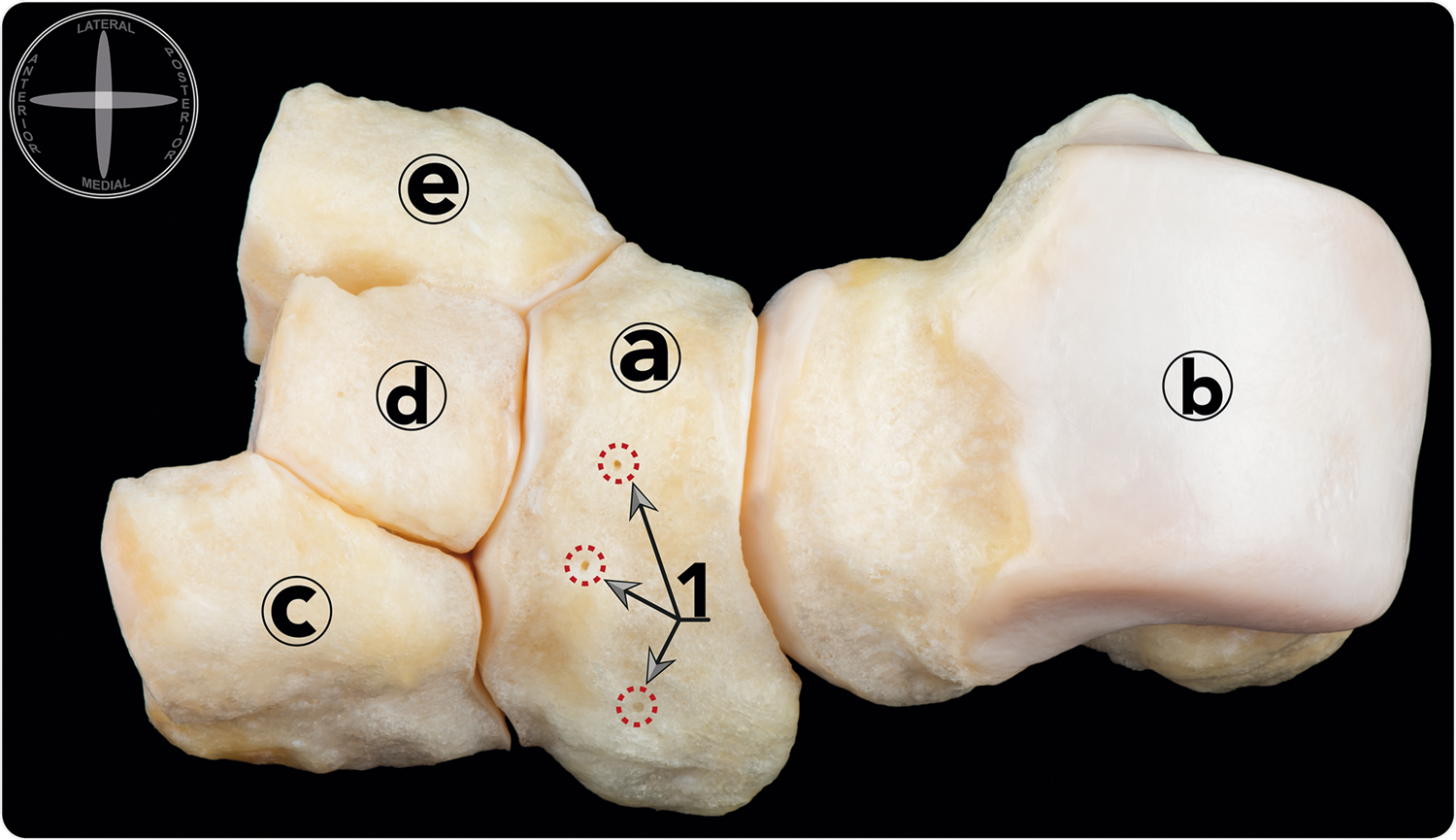
The navicular bone (a) articulates posteriorly with the talus (b), and anteriorly with the three cuneiform bones, medial (c), intermediate (d) and lateral (e). Notice the entry points of the blood vessels (branches of the dorsalis pedis artery) (1).

**Figure 2:**
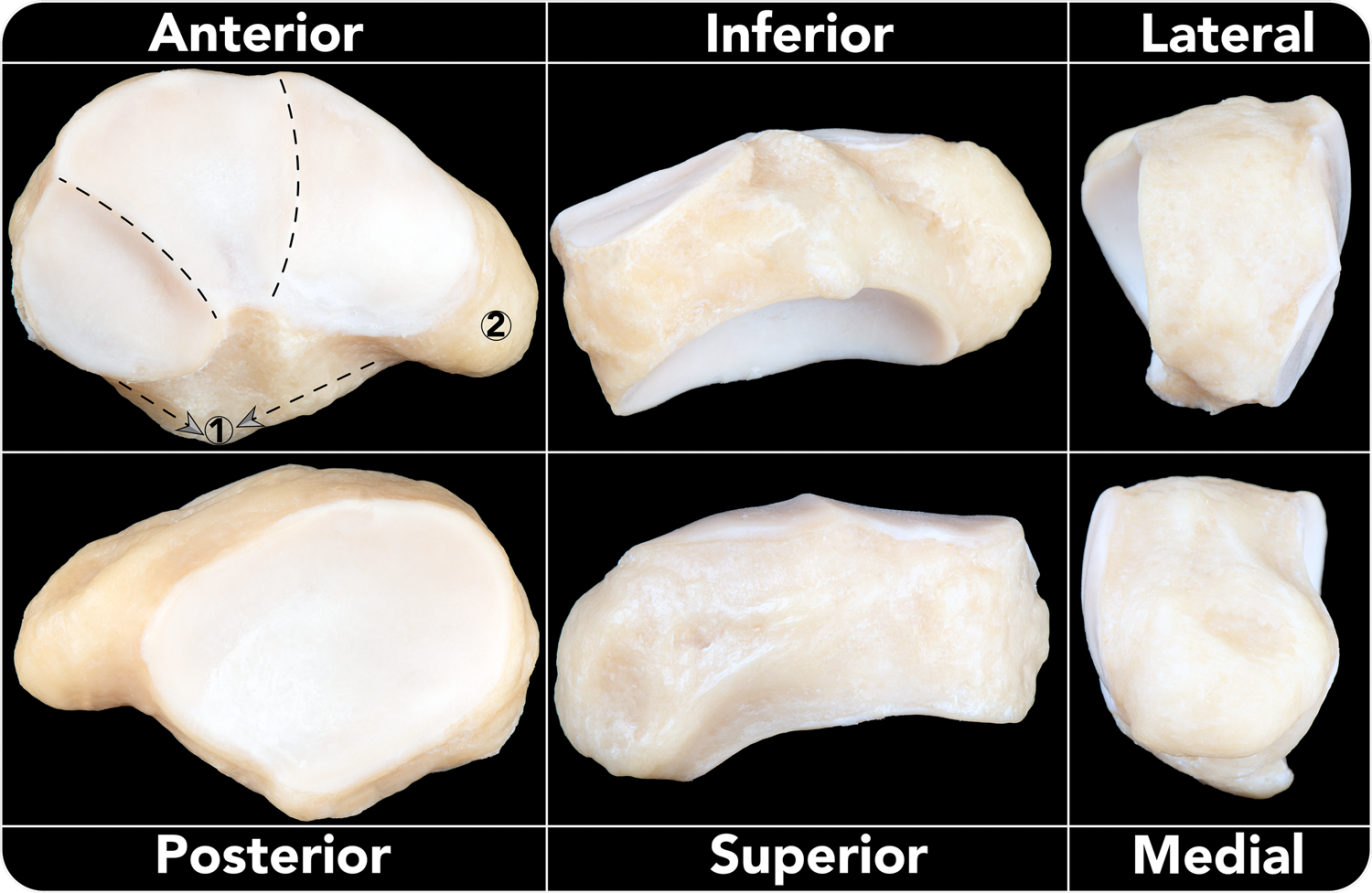
A right navicular bone seen from its six perspectives. Two subtle crests can be observed on the anterior articular surface, dividing it into three surfaces for the three cuneiform bones. On the plantar aspect, the medial and lateral plantar surfaces converge on the navicular beak (1). The navicular tuberosity is visible on the medial side (2).

The navicular is located between the talus and the three cuneiforms (Figure 1). It articulates proximally with the talar head and distally with the three cuneiforms. Its name was derived from its roughly pyriform or boat‐shaped morphology (Latin word ‘navis’) [71]. The principal oblique long axis runs in a dorsoplantar and lateromedial direction, roughly rotated 45°, to accommodate the orientation of the talar head. The rounded base corresponds to the dorsolateral region, whereas the blunted apex corresponds to the plantarmedial region. From a morphological perspective, the navicular comprises four surfaces—anterior or distal surface, posterior or proximal surface, dorsal or superior surface, and plantar or inferior surface—as well as two ends: medial and lateral (Figure 2) [23, 36].

The morphology of the navicular's posterior articular surface is biconcave, occasionally deeply concave. This surface is typically covered in articular cartilage and is most commonly described as quadrilateral, but it may also be oval, triangular, or rectangular in shape [66]. This surface articulates with the head of the talus to form an ellipsoid ball‐and‐socket joint. It does not completely encompass the talar head, permitting multi‐directional movement. The degree of concavity may be enhanced along the longitudinal axis, the transverse axis, or concurrently along both [76]. The depth of the concavity varies among individuals and may appear nearly flat in some cases [23]. Morphological alterations of the talar and navicular articular surfaces may reduce joint congruency and lead to instability, which clinically may be associated with flatfoot deformity and contribute to medial arch collapse and forefoot abduction. Key alterations in the navicular articular surface include a significant increase in concavity depth, greater cup height relative to the talar head, and a more proximally oriented navicular cup [61].

The distal surface of the navicular is typically convex or partially flat in certain regions, reniform in shape, and divided by two subtle crests into three distinct articular facets or occasionally by three to four crests into four facets, which articulate with the medial, intermediate and lateral cuneiform bones [23, 36, 42]. These crests extend from the dorsal to the plantar aspects and converge plantarly, contributing to the maintenance of the transverse tarsal arch. They delineate three articular surfaces with varying morphologies, primarily including a roughly triangular medial facet with a dorsal‐directed apex and two rounded corners at the plantar base (the largest), a triangular intermediate facet with a dorsal base and a blunted plantar‐directed apex, and a vaguely quadrilateral lateral facet (the smallest) [8, 23, 36].

The dorsal aspect of the navicular is medially broadened and laterally narrowed, with its maximal convexity corresponding to the intermediate articular facet region. At the medial end, there is a prominent blunt bony projection oriented inferomedially called the navicular tuberosity, which varies in size and is mostly pyriform in shape [66]. The plantar surface is irregular in shape and continues medially as the navicular tuberosity, which includes a bony prominence extending downwards known as the ‘navicular beak’. Both the dorsal and plantar aspects serve as attachment sites for multiple capsulo‐ligamentous structures [36, 43]. The navicular's lateral end is convex and divided into superior and inferior segments. On its superior surface, it serves as the attachment site for the powerful lateral calcaneonavicular ligament. However, its inferior surface may have a tiny articular facet for articulation with the cuboid. This facet is not consistently observed [42] and exhibits considerable morphological variability, most commonly rectangular, but also square‐shaped, triangular, quadrilateral or bean‐shaped [66].

### Soft‐tissue anatomy

The ligamentous structures surrounding the navicular are complex and play an important role in maintaining midfoot stability and the integrity of the medial longitudinal arch. The principal ligaments and associated structures include the dorsal talonavicular ligament, naviculocuneiform ligaments, naviculocuboid ligament, the spring ligament complex (SLC), lateral calcaneonavicular ligament, and the closely related tibialis posterior tendon (TPT). Together, these structures coordinate weight‐bearing and movement of the foot, ensuring optimal stability and function of the navicular.

The SLC is a complex ligamentous structure that aims to cover the space between the sustentaculum tali of the calcaneus and the navicular, therefore holding the medial side of the talar head. Globally, the SLC originates from the medial, anterior and inferior margins of the sustentaculum tali and inserts along the medial and inferior margins of the posterior articular surface of the navicular. Because it carries a fibrocartilage to articulate with a specific facet on the medial talar head, it is also referred to as the spring ligament fibrocartilaginous complex [69]. There are two main schools of thought regarding which ligaments make up this complex: some authors consider [15, 23, 36, 68] that it comprises only the superomedial calcaneonavicular ligament (SMCNL) and inferior calcaneonavicular ligament (ICNL), while other authors argue [5, 48, 59, 81, 82] that the medioplantar oblique ligament (MPOL), also referred to as the third ligament, should be included. They note that, in most cases, its fibres cannot be clearly distinguished from those of the superomedial ligament [48, 59], but in some cases, they can be entirely separated from the medial and lateral ligaments [82].

The SLC collaborates with the TPT to provide dynamic stability during standing and gait. First, injuries to this structure disrupt the biomechanical equilibrium of the foot, potentially leading to complicated medial structural disorders. Therefore, its integrity should be carefully evaluated in clinical practice, and early intervention is recommended to prevent the progression of acquired flatfoot deformity. Second, the tibiospring fascicle of the superficial deltoid ligament consistently originates from the middle portion of the anterior colliculus and inserts into the superior margin of the SMCNL as a constant structure [12], allegedly helping to maintain appropriate tension in the SLC. As a result, an injury to the superficial deltoid or specifically to the tibiospring fascicle could potentially reduce tension on the SLC, resulting in its subsequent laxity, and have biomechanical consequences on talus pronation [58]. Finally, SLC injuries increase deltoid ligament load, which may compromise medial ankle stability. In clinical practice, it is critical to properly evaluate the situations that result from the interplay of the two structures to avoid negative results caused by misdiagnosis or overlooking.

**Figure 3:**
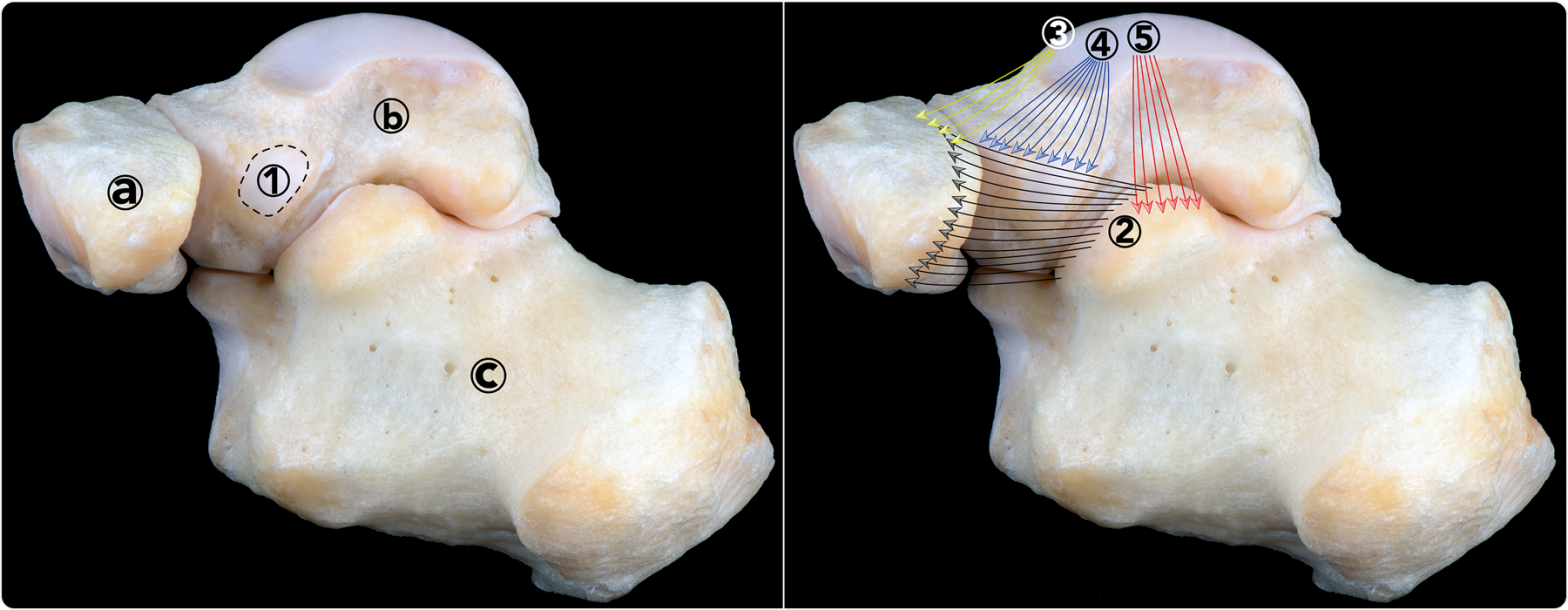
The talocalcaneonavicular joint. The head of the talus articulates both with the calcaneus and with the navicular, but part of the talar head remains uncovered by bone. That part of the talar head articulates with a fibrocartilage present in the spring ligament complex. (a) Navicular, (b) Talus, (c) Calcaneus. (1) Articular facet of the talar head for the spring ligament complex. (2) Spring ligament complex. (3) Tibionavicular fascicle of the deltoid ligament. (4) Tibiospring fascicle of the deltoid ligament. (5) Tibiocalcaneal fascicle of the deltoid ligament.

**Figure 4:**
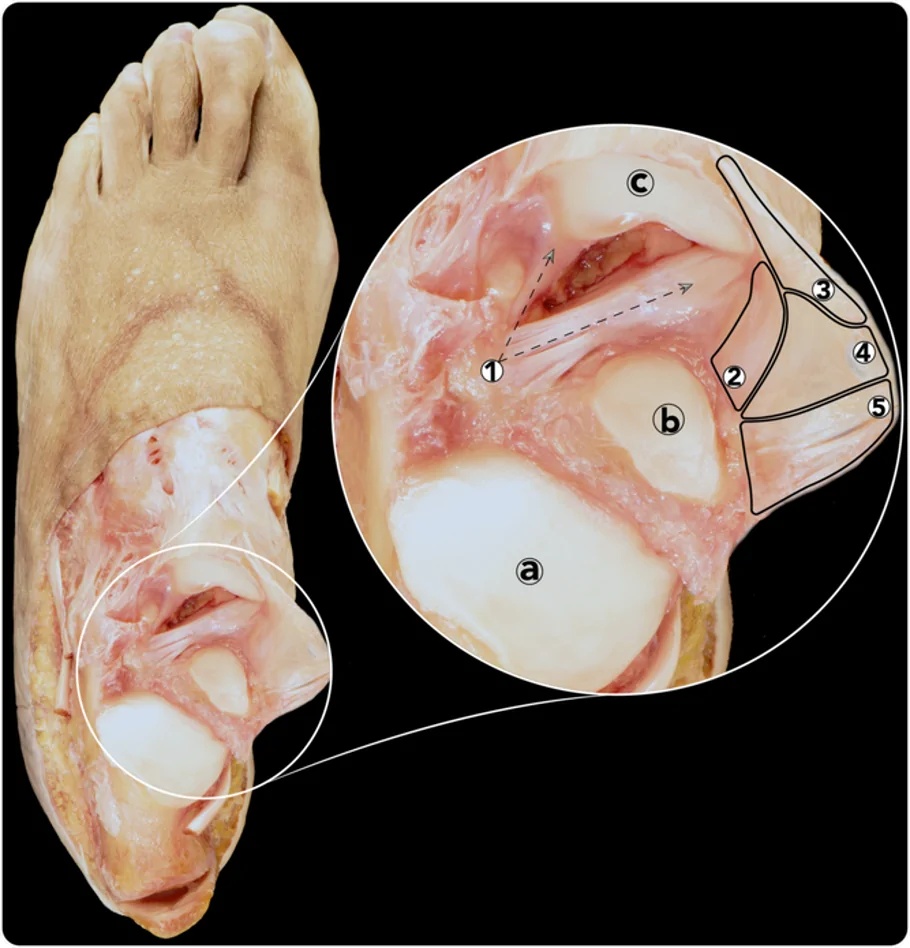
Dissection of the SLC (the talus has been removed), demonstrating how the SLC helps to create a concave cavity that accommodates the talar head. (a) Posterior subtalar articular surface of the calcaneus. (b) Anterior subtalar articular surface of the calcaneus. (c) Posterior articular surface of the navicular. (1) Inferior calcaneonavicular ligament. (2) Superomedial calcaneonavicular ligament. (3) Tibionavicular fascicle of the superficial deltoid ligament. (4) Tibiospring fascicle of the superficial deltoid ligament. (5) Tibiocalcaneal fascicle of the superficial deltoid ligament. SLC, spring ligament complex.

Regarding the SMCNL, it arises from the anteromedial margin of the middle facet of the sustentaculum tali, with its origin overlapping the insertion of the tibiocalcaneal fascicle of the superficial deltoid ligament, and inserts broadly onto the superomedial surface of the navicular [48, 59, 82]. Its fibres course anteriorly, laterally and dorsally in a sling‐like manner, with a longer superomedial margin and a shorter inferolateral margin, giving a triangular appearance when sectioned. A fibrocartilage is present in its dorsal surface, which is in turn overlaid by a single layer of synovial cells, providing a trapezoid‐shaped articular facet for the medial aspect of the talar head, which carries a complementary articular facet (Figure 3) [5, 23, 48, 59, 69, 82]. Externally, the synovial sheath of the TPT lies on top of this ligament as well as on top of the superficial deltoid, forming the path for the TPT. There is a 1–3 mm layer of loose connective tissue that is consistently observed between the SMCNL and the TPT, serving as a gliding interface (Figure 4). This layer is firmly attached to the SMCNL, and in some specimens, connecting fibres are identified between the TPT and the SMCNL in the dorsal and plantar regions [48]. Among the components of the SLC, it is the thickest, longest, and widest [59]. It typically lies in close apposition to the MPOL laterally, with no interposed fat [48, 59, 82].

The MPOL is situated between the SMCNL and the ICNL. It originates from the coronoid fossa between the anterior and middle facets of the sustentaculum tali of the calcaneus and courses anteromedially in a trapezoidal or strip‐like configuration before inserting onto the lateral aspect of the navicular tuberosity [5, 48, 55, 59]. The ICNL arises just anterolateral to the MPOL's calcaneal attachment. Its fibres are arranged in a quadrilateral pattern and oriented almost longitudinally, inserting onto the navicular beak [59, 82]. Located inferior to the talar head, it lacks fibrocartilage coverage on the dorsal surface. Of the three ligaments, it is the shortest and thinnest, with a boundary separating it from the SMCNL [59]. In clinical practice, pathological alterations of the SLC may compromise its supportive function for the talar head, leading to talonavicular joint instability and secondary changes such as adult‐acquired flatfoot deformity and joint degeneration.

**Figure 5:**
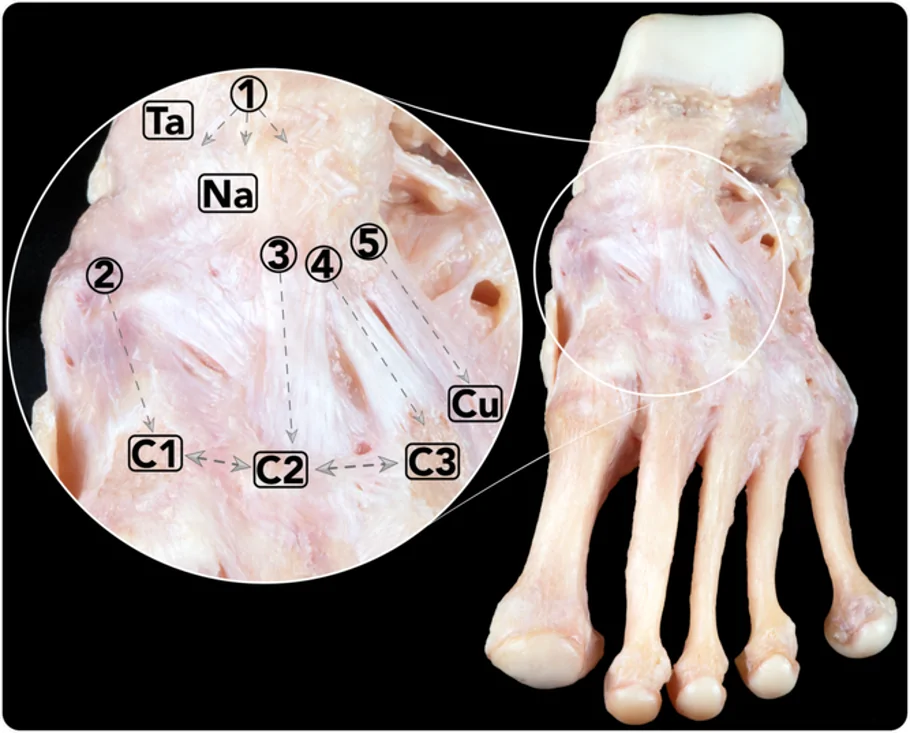
Dissection of the dorsal ligaments attaching to the navicular bone. Ta: Talus, Na: Navicular, C1: medial cuneiform, C2: intermediate cuneiform, C3: lateral cuneiform, Cu: Cuboid. (1) Dorsal talonavicular ligament. (2) Navicular‐C1 dorsal ligament. (3) Navicular‐C2 dorsal ligament. (4) Navicular‐C3 dorsal ligament. (5) Navicular‐Cuboid dorsal ligament.

The dorsal talonavicular ligament, situated in the dorsal interval between the superomedial and lateral calcaneonavicular ligaments [36], arises from the distal dorsolateral aspect of the talar neck and courses anteriorly and slightly medially to insert on the dorsal mid‐portion of the navicular (Figure 5). If extending more distally, it continues as the navicular‐intermediate cuneiform ligament. Morphologically, it is broader proximally and narrower distally, trapezoid in shape, with some fibres merging with adjacent ligaments to form a composite structure [16]. Functionally, it provides tensile resistance, and injury to this ligament compromises the dorsal stability of the talonavicular joint.

There are three dorsal and plantar naviculocuneiform ligaments, which connect the navicular to each of the three cuneiforms (Figure 5). They primarily reinforce the naviculocuneiform joint, thereby contributing to joint stability and maintaining the medial longitudinal arch [80]. According to their anatomical locations, they are divided into two groups: dorsal and plantar ligaments. On one hand, the dorsal ligaments originate from the dorsal aspect of the navicular and extend distally as multiple fibre bundles, rather than a single band, inserting onto the dorsal surfaces of the respective cuneiforms. Among them, the first naviculocuneiform ligament is the most robust [36]. On the other hand, the first plantar naviculocuneiform ligament is a continuation of the TPT after its convergence on the plantar and medial aspects of the navicular. It is a robust fibrous band, with the greatest thickness on the plantar surface. Its longitudinal fibres attach to multiple surfaces of C1 in a saddle‐like configuration. In most specimens, it appears as a direct extension of the TPT, while in others it presents as a distinct ligament [80]. The second and third plantar naviculocuneiform ligaments originate from the plantar surface of the navicular, located between the navicular tuberosity and the navicular beak, and insert onto the posterior portion of the corresponding cuneiform crest [36].

**Figure 6:**
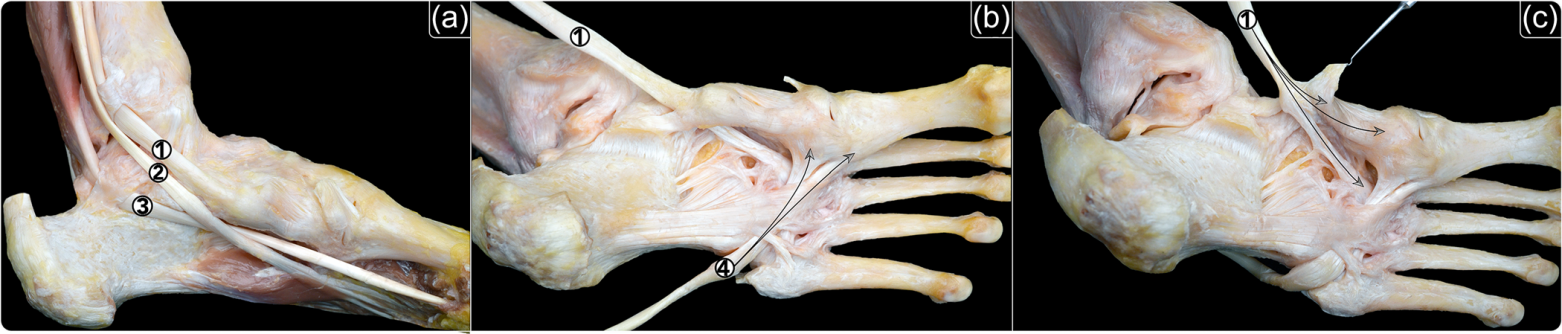
Tibialis posterior tendon (TPT) insertion on the navicular bone and its expansions, enabling the TP to be the main active stabilizer of the medial longitudinal arch of the foot. (a) Medial view. (b) Plantar view. (c) Plantar view with TPT expansions, inserting on the navicular, on the plantar naviculo‐cuneiform ligament, and on the second and third metatarsal bases. (1) TPT. (2) Flexor digitorum longus tendon. (3) Flexor hallucis longus tendon. (4) Peroneus longus inserting in first metatarsal base and medial cuneiform.

The TPT originates from the tibia, fibula, and interosseous membrane and courses posterior to the medial malleolus beneath the flexor retinaculum. Its primary attachment sites are the navicular bone, the medial cuneiform (C1), and the lateral cuneiform (C3) [7, 51, 57, 87, 92]. The main tendon only inserts onto the navicular bone and the C1 [57], with its principal footprint located on the plantar aspect of the navicular [92]. At this site, three distinct insertional morphologies have been described: crescent‐shaped (14.6%), oval‐shaped (75.6%), and trapezoid‐shaped (9.8%) [92]. In addition, accessory slips may extend to the cuneiforms, cuboid, bases of the metatarsals, and occasionally the calcaneus (Figure 6). Beyond its osseous insertions, some accessory slips may also attach to several plantar soft tissues, including the plantar calcaneonavicular ligament, flexor hallucis brevis, abductor hallucis and the fibularis longus tendon [7, 38, 39, 51, 57]. Notably, these distal insertional footprints, particularly those of the additional slips, exhibit considerable morphological variability [7, 39, 51, 57, 87, 92], which may vary by race, ethnicity, and sex [57]. Based on the number of tendon insertions, the TPT can be classified into four major types: Type 1 (single insertion), Type 2 (double insertions), Type 3 (triple insertions) and Type 4 (quadruple insertions). However, Types 3 and 4 are further subdivided into various subtypes according to the insertion sites of accessory tendons [51, 57]. Among them, the most common type is reported to be either Type 3 [51] or Type 4 [57]. These findings indicate that the multiple tendinous slips and insertional footprints of the TPT provide firm anchorage to the plantar aspect of the foot. Functionally, the TPT is a key dynamic stabilizer of the medial longitudinal arch. Clinically, its degeneration or injury is one of the main causes of midfoot collapse and adult‐acquired flatfoot deformity [72]. Furthermore, traction forces generated by the TPT and the spring ligament impart additional mechanical load to the navicular.

### Anatomical factors influencing navicular stress fractures

Navicular stress fractures have an unclear aetiology that is probably caused by a combination of extrinsic and intrinsic causes [2, 6, 21, 46, 47, 60, 63, 74, 93]. The foremost potential anatomical factor is the uneven vascular distribution of the navicular bone. The central region of the navicular bone exhibits an avascular zone or hypovascular region, with little to no direct vascular penetration, and is subjected to high shear stress [28]. In this region, microinjuries are prone to accumulation due to poor healing capacity, ultimately disrupting bone remodelling homoeostasis.

**Figure 7:**
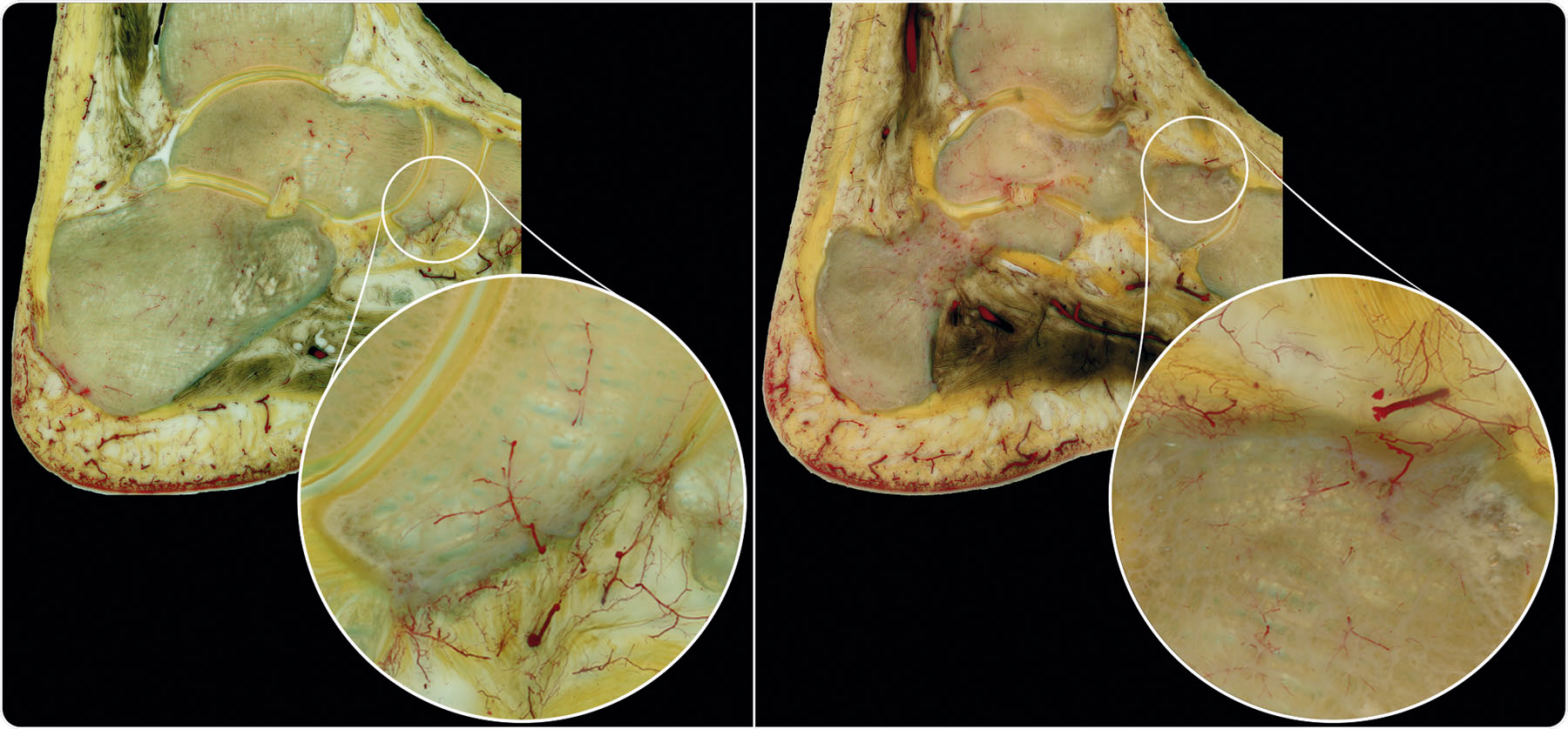
Sagittal cross‐section of specimens injected with red resin and then plastinated, to demonstrate the blood supply of the navicular bone. Left: Branch of the medial plantar artery entering the plantar part of the navicular from the spring ligament complex. Right: Branch of the dorsalis pedis artery entering the dorsal part of the navicular.

The navicular bone receives its blood supply from several consistent vascular branches, with the majority of its dorsal and medial regions supplied by multiple nutrient arteries arising from the medial tarsal branches of the dorsalis pedis artery, as well as direct branches from the anterior tibial artery. The lateral region is supplied by the distal lateral tarsal artery, another branch of the dorsalis pedis artery, while the medial plantar aspect receives blood from the superficial branch of the medial plantar artery, which originates from the posterior tibial artery (Figure 7) [22, 46]. In another study, it was found that the distribution of vascular foramina in the navicular bone is markedly uneven. They are primarily located on the non‐articular dorsal surface (the most abundant), followed by the plantar and lateral surfaces, with the medial surface having the fewest or even no foramina. Furthermore, there are no vascular foramina at all on the anterior or posterior articular surfaces. These findings reveal a distinct variation in the vascular supply of the navicular bone, with the medial and central regions exhibiting the poorest perfusion, which may affect the bone's capacity for healing and remodelling [63]. Therefore, the limited blood supply in specific regions impairs the capacity for microdamage repair. If repetitive micro‐injuries persist and exceed the bone's intrinsic limited healing potential, micro‐injuries accumulate, and this can ultimately progress to stress fractures.

From a surgical perspective, both reduction and fixation should aim to minimize periosteal stripping, particularly around the nutrient foramen, to avoid compromising the bone's vascular supply. Considering the vascular anatomy of the navicular and that dorsal plating requires periosteal elevation, we could hypothesize that this might disrupt the dorsal blood supply entering the bone and subsequently impair healing. In contrast, screw fixation better preserves the periosteal tissue and could therefore better maintain the native vascular integrity of the navicular and potentially promote faster bone union.

Within the intraosseous vascular pattern, a relatively avascular area is present in the central dorsal third of the navicular bone in a small proportion of individuals, which may contribute to this region's stress fractures or delay the healing process of such injuries [23, 46] and may increase the incidence of complications and also make return to sport more difficult [27, 44, 47]. Moreover, based on their vascular anatomy, certain individuals may have an increased risk of developing navicular stress fractures. However, in most navicular bones (58.8%), a dense intraosseous vascular supply is present without an avascular zone [46], implying that biomechanical or other clinical factors may have a bigger impact on the stress fractures.

Another crucial biomechanical factor is the presence of transverse tensile strain, longitudinal compressive forces and lateral shear forces in the navicular [40, 50]. On the one hand, finite element simulations of the foot‐ankle during normal standing and the mid‐stance phase of gait revealed that the von Mises stress peaks in the navicular and the C3 were higher than those in the C1 and C2 [10, 50]. Among all midfoot bones, the navicular endures the highest first principal stress (tensile stress) and the lowest absolute value of the third principal stress (compressive stress), with its higher stress concentration primarily attributed to the influence of the spring ligament [50] and the TPT [28]. Given that the tensile strength of cancellous bone is around 70% of its compressive strength [4, 35], making it more susceptible to tensile rather than compressive failure. This biomechanical property explains the greater stress fracture risk of the navicular. On the other hand, in vitro experiments showed that both the navicular and the C1 were subjected to compressive strain in the longitudinal direction. The former exhibited the highest compressive stresses on the articular surfaces of the talonavicular and cuneonavicular joints. Finally, the navicular is additionally subjected to lateral shear forces from the second ray [40]. Due to its central position within the midfoot skeletal chain and the complex force transmission it endures, the navicular is subjected to longitudinal compressive stress, transverse tensile strain and lateral shear forces. These combined stresses concentrate in the narrower and weaker mid‐region of the navicular bone, which is prone to cause local microdamage [50]. This further explains why the middle third of the navicular is most vulnerable to stress fractures under recurrent loading. On physical examination, this location also correlates to the ‘N’ spot, which is the characteristic site of tenderness in a navicular fracture [28, 85].

Summarizing, the navicular is an important bone in the midfoot, part of the midtarsal joint. As such, it serves as a crucial mechanical link within the medial longitudinal arch, facilitating force transfer between the hindfoot and forefoot. Its susceptibility to pathology, such as AVN, can be attributed to its unique anatomical challenges: an inconsistent vascular network that is poorly documented across the literature and a complex geometry that must sustain a combination of tensile and compressive loads. This review suggests that navicular AVN is not a simple consequence of ischaemia but a multifactorial disorder. The initial hypothesis of primary vascular insufficiency is now better explained by a cumulative stress model, where anatomical foot shape, repetitive high‐impact stress, nutritional factors (including vitamin D levels) and pre‐existing vascular issues collectively overwhelm the bone's reparative capacity.

## PART 2: THE BASE OF THE FIFTH METATARSAL

Stress fractures of the proximal region of the M5 are among the most common lower extremity overuse injuries, especially among runners, basketball players, football players and others who participate in high‐impact activities [25, 26, 32, 37, 70, 78]. These fractures frequently have an insidious beginning with nonspecific symptoms, such as vague lateral foot discomfort, and are easily misdiagnosed or delayed. Due to the unique vascular anatomy of the proximal M5 [45, 75, 77], delays in treatment may lead to a high incidence of complications, including delayed union, nonunion, and refracture [94]. The complex osseous anatomical configuration of the proximal M5, combined with its biomechanical loading characteristics and its close relationship to surrounding soft tissue structures [18, 33, 73, 83], such as the peroneus brevis tendon (PBT), the peroneus tertius tendon (PTT) and the lateral band (LB) of the plantar aponeurosis, make this region particularly susceptible to concentrated stress and traction forces. As a result, the proximal M5 is a frequent site of stress‐related injury. The primary objective of this article part is to elucidate the anatomical characteristics of the proximal M5, enhance understanding of the fracture patterns, inform preventive strategies in high‐risk populations, and guide the clinical management of patients with fractures [28].

### Bony anatomy

**Figure 8:**
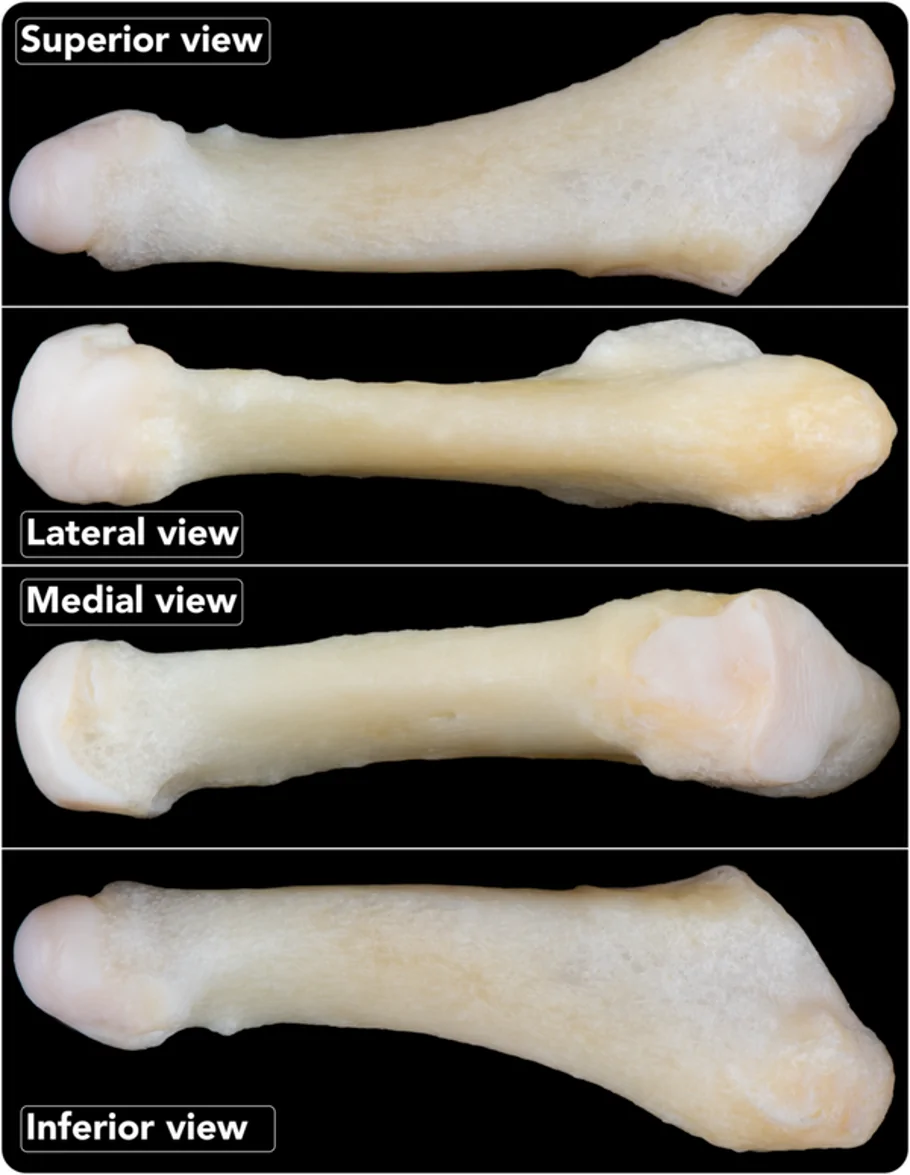
Anatomy of the fifth metatarsal bone, seen from its four perspectives.

Anatomically, the M5 is divided into five sections: base, metaphyseal–diaphyseal junction, shaft or diaphysis, neck and head (Figure 8). The base, found at the proximal end, consists of a regionally enlarged tuberosity (formerly known as the styloid process, but that name is no longer in use) [90], projecting towards the plantar‐lateral and posterior aspect, as well as an articular surface that articulates proximally with the cuboid [13, 36, 41]. This region is often triangular [18] or quadrangular in shape and acts as attachment points for several tendinous structures [18, 33, 73, 83]. Given these anatomical characteristics, the base is the most common site of avulsion fractures. The shaft that links the base and neck of the M5 is a thin, slightly plantarly curved structure with a thick cortical bone [36]. It plays a critical role in load transmission and is the most common site for stress fractures due to repetitive mechanical loading. There exists an anatomical transitional region between the base and the shaft of the M5, called the metaphyseal–diaphyseal junction. This area is precisely located between the proximal and distal margins of the articular surfaces of the fourth to fifth intermetatarsal joint and corresponds anatomically to the region of Jones fractures [41, 73]. The M5's distal part is represented by the head. It has a roughly spherical shape, with the articular surface oriented distally and medially, forming a joint with the base of the proximal phalanx of the fifth toe [36].

**Figure 9:**
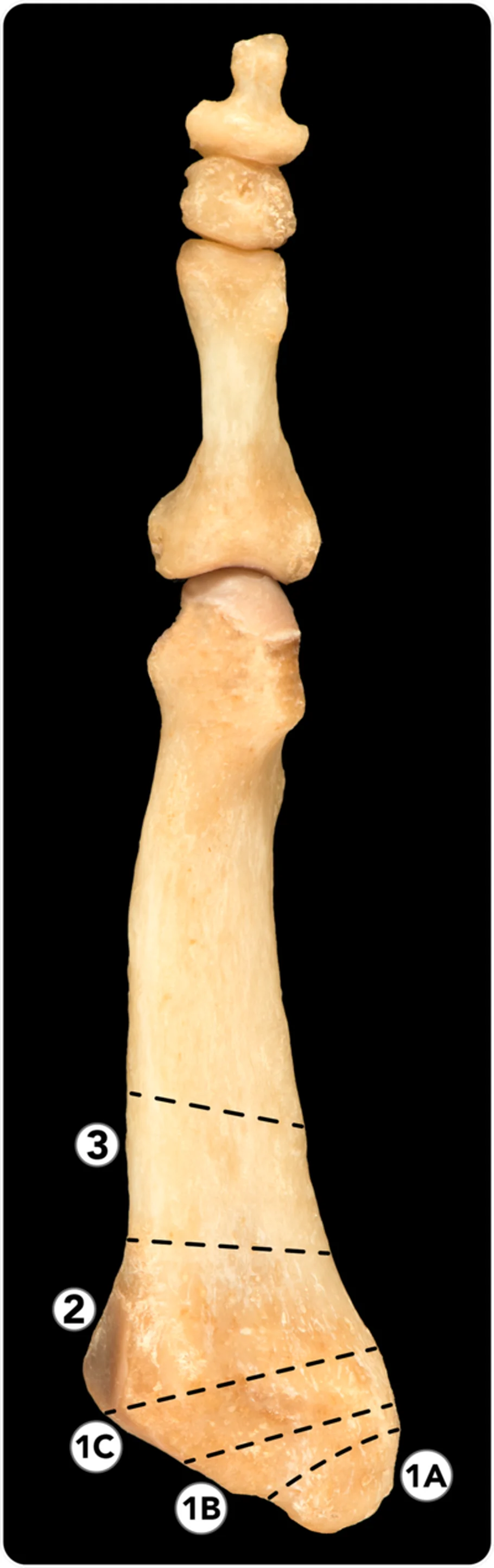
The fifth metatarsal and fifth toe depicting the fracture zone as shown by DeVries et al. Zone 1: tuberosity of the fifth metatarsal; Zone 2: Jones fracture. Zone 3: Stress fracture. Additionally, zone 1 can be subdivided into three zones: 1A: attachment of the lateral band of the plantar aponeurosis. 1B: attachment of the peroneus brevis tendon. Zone 1C: posterior articular facet of the fifth metatarsal with the cuboid bone.

The proximal region of the M5 exhibits distinct differences in bony architecture, vascular supply [45, 75, 77], biomechanical loading and various tendon attachments [18, 33, 73, 83] across its subregions, all of which critically influence fracture healing potential and treatment strategies [9, 13, 41, 84]. This region can be further subdivided into three anatomically and clinically relevant zones by Lawrence and Botte [41]: the tuberosity region (avulsion fractures zone), the metaphyseal–diaphyseal junction (Jones fracture zone), and the proximal 1.5 cm of the diaphysis, which is typically associated with stress fractures. Based on the anatomical classification, two perpendicular lines to the long axis of the M5 are drawn using the proximal and distal ends of the fourth to fifth intermetatarsal joint as reference points. These two lines divide the proximal portion of the M5 into three distinct zones (Figure 9): Zone 1 (avulsion fractures zone): lies proximal to the first (proximal) line; Zone 2 (Jones fracture zone): occupies the region between the two lines; Zone 3 (proximal diaphyseal stress fractures zone): encompasses the distal 1.5 cm beyond the second (distal) line [11, 13, 41, 73].

Based on the different attachment sites of the PBT and the LB of the plantar aponeurosis in the tuberosity region, DeVries et al. [18] proposed a further subdivision of Zone 1 (base of the M5) into three anatomical zones (Figure 9). Zone A: The attachment area for the LB of the plantar aponeurosis, located on average 6.6 ± 2.2 mm from the inferior border, 9.5 ± 2.9 mm from the proximal edge, and 11.5 ± 0.9 mm from the lateral aspect. This zone is non‐articular. Zone B: The attachment site for the PBT, situated on average 12.0 ± 2.2 mm from the inferior border, 10.2 ± 2.2 mm from the proximal edge and 11.5 ± 0.9 mm from the lateral aspect. This region is also non‐articular. Zone C: The posterior articular surface of the proximal M5 with the cuboid bone. Avulsion fractures involving different anatomical zones determine whether dynamic muscular traction is present and whether the articular surface is involved. They differ in terms of fracture stability and surgical requirements, providing important guidance for clinical decision‐making.

The proximal M5 has two distinct articular facets: the intermetatarsal articular surface for M4 and the posterior articular surface for the cuboid bone. They are separated by a blunt crest. The surface for M4 is located on the medial side of Zone 2. Thus, Jones fractures are highly likely to involve injury to the articular cartilage, except when the fracture extends into the extreme distal portion of the joint. This is because the fourth to fifth intermetatarsal joint is not entirely articular and contains both articular and fibrous components. The articular cartilage progressively tapers from proximal to distal, with the joint becoming increasingly fibrous towards its distal extent [64]. The intermetatarsal articular surface of M5 has an average articular surface area of 143 ± 30 mm^2^, with approximately 48% of this surface covered by flat articular cartilage, measuring on average 9.5 ± 1.2 mm in length [64]. To prevent iatrogenic cartilage injury, screw placement should avoid penetrating this region. Two distinct morphological variants of the joint surface were observed: oval and triangular [36, 64]. The oval variant, which was the most common, featured centrally located articular cartilage surrounded by a rim of fibrous tissue. In contrast, the triangular variant had a vertical proximal base with an apex directed plantar‐distally. In this form, the fibrous component was located dorsal to the articular cartilage [64].

The medial aspect of the M5 base also includes its posterior articular surface and the adjacent non‐articular region. This posterior articular surface is dedicated to the anterior‐lateral articular surface of the cuboid, and is approximately triangular in shape, with its base oriented medially and apex directed laterally [17, 36]. The articular surface has a mean width and height of 15.2 ± 2.4 and 13.7 ± 2.3 mm, respectively, while the adjacent irregular non‐articular region measures 11.5 ± 0.9 mm in width and 12.4 ± 1.7 mm in height on average [18].

### Vascular

**Figure 10:**
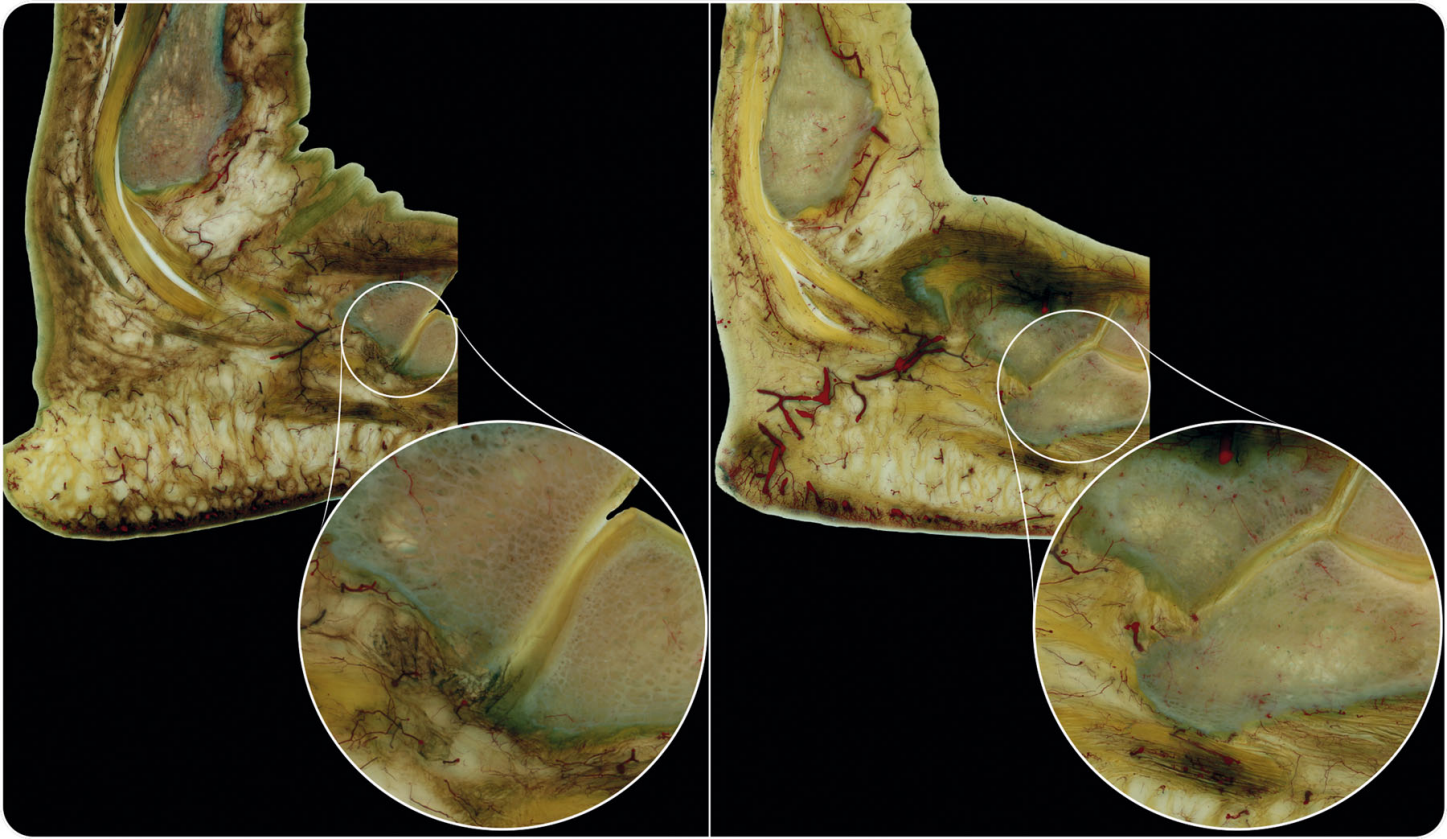
Sagittal cross‐section of specimens injected with red resin and then plastinated, where the morphology of the fifth metatarsal base can be observed in detail. Left: the joint line of the base with the cuboid can be observed. Right: some blood vessels can be observed entering the tuberosity, with a central area with less supply. The joint line with the cuboid and with the fourth metatarsal is visible.

The blood supply of the M5 primarily derives from branches of both extraosseous and intraosseous vessels, with a notably uneven distribution across its anatomical regions [45, 75, 77]. The base is characterized by a rich vascular network, conferring a strong healing potential. In contrast, the metaphyseal–diaphyseal junction and the proximal diaphysis receive relatively limited blood flow, creating a hypovascular zone that predisposes fractures in this region to delayed union or nonunion (Figure 10) [77]. This unique vascular pattern is therefore a critical factor in guiding treatment strategies and predicting clinical outcomes.

The extraosseous blood supply of the M5 consists of three main sources [36, 45, 75]. The lateral tarsal artery arises from the dorsalis pedis artery, and its terminal branches form an arterial network with small branches of the lateral plantar artery and terminal branches of the peroneal arteries around the proximal end of the M5; the plantar metatarsal arteries, arising from the deep plantar arch formed by the lateral plantar artery of the posterior tibial artery and the deep branch of the dorsalis pedis artery; and the fourth metatarsal plantar artery, a direct branch of the lateral plantar artery, travels along the medial diaphysis of the M5. Meanwhile, the extraosseous vessels form an anastomosis at the proximal end of the joint between the M4 and M5.

The intraosseous blood supply of the M5 can be classified into three principal components [45, 75, 77]. First, the periosteal plexus, derived from branches of the dorsal and plantar source arteries, forms a circumferential network around the M5 and gives rise to penetrating branches that pierce the cortex. Second, the metaphyseal arteries, as the terminal branches of the lateral tarsal artery, penetrate the proximal epiphysis of M5 and follow a randomly distributed course within the bone. This vascular pattern provides abundant intraosseous blood supply to the tuberosity, making fractures in this region less likely to disrupt perfusion and therefore demonstrating strong healing potential. Finally, a nutrient artery arising from the fourth plantar metatarsal artery penetrates the cortex at the junction of the proximal and middle thirds on the medial side of the M5. It then branches into multiple smaller vessels that extend towards both the proximal and distal ends to supply the bone. This arterial distribution exhibits a distinctive linear pattern and proximally terminates near the metaphyseal–diaphyseal junction. Therefore, in the proximal diaphysis of the M5, the intramedullary vascular pattern and its proximity to the nutrient foramen make fractures in this region highly susceptible to injuring or even disrupting the intraosseous blood supply and creating a potential relative avascular zone, which can affect fracture healing.

### Soft‐tissue anatomy

**Figure 11:**
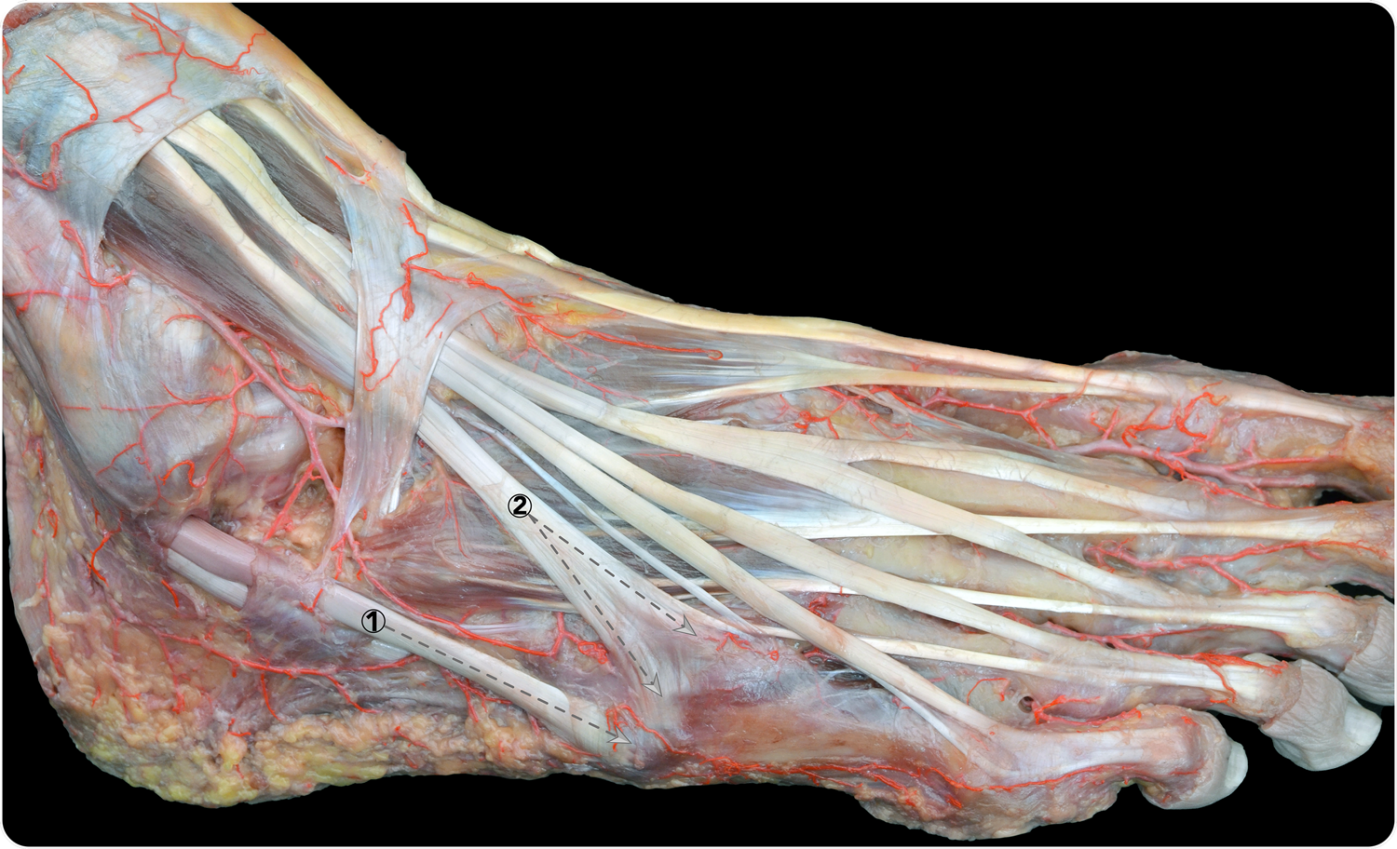
Two tendons insert in the fifth metatarsal tuberosity and are therefore involved in the fracture patterns on this structure. (1) Peroneus brevis. (2) Peroneus tertius, which also inserts in the base of the fourth metatarsal.

**Figure 12:**
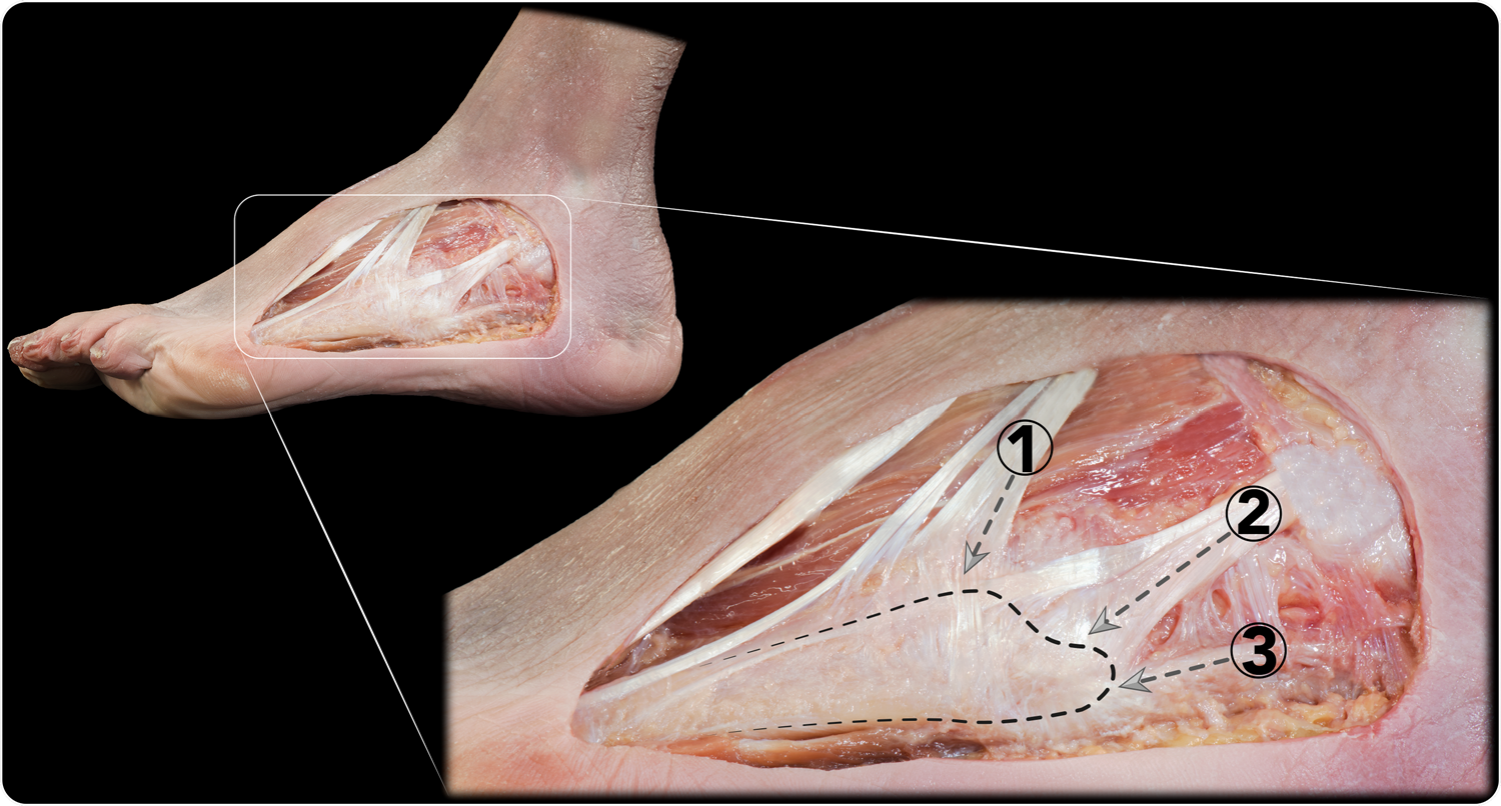
Anatomical dissection of the lateral foot showing the three anatomical structures inserting in the fifth metatarsal. (1) Peroneus tertius tendon, (2) peroneus brevis tendon and (3) lateral band of the plantar aponeurosis.

The PBT, whose attachment is typically oval‐shaped, inserts onto the dorsolateral, non‐articular region of the M5 tuberosity [13, 18, 29] and is consistently present (Figure 11) [33]. Its insertion measures approximately 3.5 ± 1.0 mm in width and 5.2 ± 1.2 mm in height, with an attachment length of approximately 10.2 ± 2.2 mm [18]. In fact, the attachment sites of the PBT and the LB are not entirely separate; rather, they gradually join, forming a common attachment at the tuberosity (Figure 12). In some anatomical variants, the distal partial tendon fibres of the PTT (which also insert in the base of M4) may extend to insert at the base of the M5, forming an intertendinous connection with the PBT [20, 30]. As a result, in individuals with this anatomical connection, both the PBT and PTT may contribute synergistically to certain avulsion fracture patterns. However, Kaneko et al. [33] reported that the PBT and the LB differ in both their attachment regions and the number of insertion sites. The PBT (40.2%) and LB (26.5%) attach proximal to Zone 2, whereas the remaining attachment sites extend into Zone 2 or even reach Zone 3. In contrast, the PTT is exclusively attached distally within Zone 1. The PBT primarily inserts onto the base of the M5. In some cases, additional slips were observed [24, 30, 65], extending to the extensor of the fifth toe, to both the extensor of the fifth toe and the M4, reattaching to the base of the M5, or inserting onto the diaphysis of the M5 [24, 30]. The combined attachment patterns of all three structures may collectively influence traction stress. Furthermore, the spatial relationship between these attachment sites and the fracture line may play a critical role in determining fracture stability and healing potential [33, 49, 83].

The LB of the plantar aponeurosis attaches to the inferolateral, non‐articular side of the M5 tuberosity [13, 18, 83] and is always present (Figure 12) [33]. This attachment is also oval and located inferior and posterior to the insertion of the PBT. The average width, height and length of the attachment are 4.0 ± 0.9, 5.5 ± 1.4 and 9.5 ± 2.9 mm, respectively [18].

The PTT also inserts onto the base of M5. Previously believed to be an anatomical variant, it is now clearer that it is a constant muscle that only occasionally is not present. It originates from the distal half of the fibula [95] and inserts into the distal dorsal surface of the base of the M4 and M5, either in a band‐shaped or fan‐shaped form (most common) [34, 52, 53]. Its exact insertion onto M5 is located distal to the insertion sites of both the PBT and the LB [13, 18, 83], and its prevalence in the literature has been reported to be 88.2% [83], 89.5% [31], 93% [79], 93.75% [89], 95% [14] and 100% [1], respectively. However, its presence still shows considerable variability and is inconsistent in different ethnicities and populations [52, 62, 79]. The PTT may present as either a single or double fascicle, exhibiting highly variable morphological patterns [20, 31, 34, 52, 54, 79, 96, 97]. In some rare instances, an accessory or duplicated PTT may be observed [1]. Most insertions are located on the M5 [20, 31, 52, 89]. Among these, the most common configuration consists of a single fascicle inserting either onto the dorsal base of the M5 (70%) [89], (50%) [31], (84.8%) [1], 54.8% [20] or onto its shaft (45%) [19]. Other variations include insertion on both the M4 and M5 [20, 89]; a single slip attaching broadly to the base of the M5; bifurcated slips attaching to both the base and shaft of the M5 [3, 34, 52]; and bifurcated slips inserting onto the bases of both the M4 and M5. In rare instances, it may fuse with the PBT and terminate at the base of the M5 [52].

#### Biomechanics

The proximal M5 is subjected to complex loading patterns, including torsional, bending, and tensile stresses, which not only contribute to the development and progression of stress fractures but also affect fracture stability [19, 56, 67, 88]. On the one hand, the lateral side of the proximal Zone 2 and Zone 3 of the M5 is subjected to torsional loading, which results in internal rotation of the proximal segment relative to the distal segment. Such torsional stresses may contribute to the progression of stress fractures and, once a fracture has occurred, may cause rotational displacement of the fracture fragments. However, the tendinous forces of the PBT and PTT are likely to generate these torsional moments [88]. On the other hand, the M5 is subjected to bending moments along its longitudinal axis, with the greatest moments occurring during acceleration manoeuvres, followed by straight running. This suggests that acceleration is a high‐risk activity for M5 stress fractures. Consequently, increasing acceleration manoeuvres or running distance during training may affect the balance between bone resorption and formation, thereby increasing the risk of fracture [19, 56]. Finally, Morris et al. [49] demonstrated that the PBT exerts a deforming force on the proximal fragment in proximal M5 fractures. This deforming force was more pronounced in simulated Jones fractures than in simulated avulsion fractures. This finding indicates that fractures distal to the PBT insertion are significantly less stable than those proximal to it. Meanwhile, the PBT is also a source of postoperative instability following Jones fracture fixation [91]. Richli and Rosenthal [67] found that transverse fractures at the base of the M5 are predominantly caused by avulsion from the LB, rather than by the PBT. When the LB is intact and subjected to tension in plantarflexion combined with inversion, transverse fractures at the M5 base occur. However, once the LB is transected, the fracture can no longer be induced, even if the PBT remains intact.

In summary, the M5 constitutes a key element of the lateral column of the forefoot and is an integral component of the lateral longitudinal arch, facilitating force transmission from the hindfoot to the distal forefoot. Its proximal region, owing to unique osseous and soft tissue anatomy, is subjected to complex biomechanical loading during activity, including torsion, bending and tensile forces, making it particularly prone to stress‐related injuries. Furthermore, the distinct vascular patterns within different regions of the proximal M5 result in marked variability in healing potential, thus requiring the development of personalized treatment strategies. Consequently, injury and recovery of this region reflect a combined influence of biomechanical stresses and vascular supply.

## AUTHOR CONTRIBUTIONS

Xiuqi Liu and Choon Chiet Hong wrote most of the manuscript, Francisco Reina and Anna Carrera wrote the surgical approach part, Enric Alcolea‐Madera helped in the preparation of the specimens for the figures, Jorge Javier Del Vecchio did two revisions of the text, Miki Dalmau‐Pastor did four revisions of the manuscript and prepared all the figures.

## CONFLICT OF INTEREST STATEMENT

The authors declare no conflicts of interest.

## ETHICS STATEMENT

The authors have nothing to report.

## Data Availability

Data sharing is not applicable—no new data generated, or the article describes entirely theoretical research.

## References

[1] Afroze MKH , Muralidharan S , Ebenezer JL , Muthusamy S . Morphological variations of peroneus tertius: a cadaveric study with anatomical and clinical consideration. Medeni Med J. 2020;35(4):324–329. 33717625 10.5222/MMJ.2020.98512 PMC7945728

[2] Barrack MT , Gibbs JC , De Souza MJ , Williams NI , Nichols JF , Rauh MJ , et al. Higher incidence of bone stress injuries with increasing female athlete triad‐related risk factors: a prospective multisite study of exercising girls and women. Am J Sports Med. 2014;42(4):949–958. 24567250 10.1177/0363546513520295

[3] Bauer SA , Kandathil SA , Hirtler L . Peroneus tertius revisited: the morphological variability of the bifurcated peroneus tertius insertion—an anatomical study. Ann Anat. 2023;250:152164. 37804928 10.1016/j.aanat.2023.152164

[4] Bayraktar HH , Morgan EF , Niebur GL , Morris GE , Wong EK , Keaveny TM . Comparison of the elastic and yield properties of human femoral trabecular and cortical bone tissue. J Biomech. 2004;37(1):27–35. 14672565 10.1016/s0021-9290(03)00257-4

[5] Belis AM , Foote GA . The spring ligament complex‐anatomy and function. Clin Podiatr Med Surg. 2022;39(3):393–403. 35717057 10.1016/j.cpm.2022.02.003

[6] Bennell K , Matheson G , Meeuwisse W , Brukner P . Risk factors for stress fractures. Sports Med. 1999;28(2):91–122. 10492029 10.2165/00007256-199928020-00004

[7] Bloome DM , Marymont JV , Varner KE . Variations on the insertion of the posterior tibialis tendon: a cadaveric study. Foot Ankle Int. 2003;24(10):780–783. 14587993 10.1177/107110070302401008

[8] Borrelli G , Albiero M , Jastifer J . Anatomy of the naviculocuneiform joint complex. Foot Ankle Orthop. 2024;9(2):24730114241245396. 38601321 10.1177/24730114241245396 PMC11005504

[9] Cheung CN , Lui TH . Proximal fifth metatarsal fractures: anatomy, classification, treatment and complications. Arch Trauma Res. 2016;5:e33298. 28144601 10.5812/atr.33298 PMC5251206

[10] Cheung JT‐M , Zhang M , Leung AK‐L , Fan Y‐B . Three‐dimensional finite element analysis of the foot during standing—a material sensitivity study. J Biomech. 2005;38(5):1045–1054. 15797586 10.1016/j.jbiomech.2004.05.035

[11] Chloros GD , Kakos CD , Tastsidis IK , Giannoudis VP , Panteli M , Giannoudis PV . Fifth metatarsal fractures: an update on management, complications, and outcomes. EFORT Open Rev. 2022;7(1):13–25. 35073515 10.1530/EOR-21-0025 PMC8788151

[12] Dalmau‐Pastor M , Malagelada F , Guelfi M , Kerkhoffs G , Karlsson J , Calder J , et al. The deltoid ligament is constantly formed by four fascicles reaching the navicular, spring ligament complex, calcaneus and talus. Knee Surg Sports Traumatol Arthrosc. 2024;32(12):3065–3075. 38757967 10.1002/ksa.12173 PMC11605026

[13] Dameron T . Fractures and anatomical variations of the proximal portion of the fifth metatarsal. J Bone Joint Surg. 1975;57(6):788–792. 1099103

[14] Das S , Haji Suhaimi F , Abd Latiff A , Pa Pa Hlaing K , Abd Ghafar N , Othman F . Absence of the peroneus tertius muscle: cadaveric study with clinical considerations. Rom J Morphol Embryol. 2009;50(3):509–511. 19690784

[15] Davis WH , Sobel M , DiCarlo EF , Torzilli PA , Deng X , Geppert MJ , et al. Gross, histological, and microvascular anatomy and biomechanical testing of the spring ligament complex. Foot Ankle Int. 1996;17(2):95–102. 8919408 10.1177/107110079601700207

[16] De Dea M , L Loizou C , Allen GM , Wilson DJ , Athanasou N , Uchihara Y , et al. Talonavicular ligament: prevalence of injury in ankle sprains, histological analysis and hypothesis of its biomechanical function. Br J Radiol. 2017;90(1071):20160816. 27993094 10.1259/bjr.20160816 PMC5601506

[17] De Palma L , Santucci A , Sabetta SP , Rapali S . Anatomy of the Lisfranc joint complex. Foot Ankle Int. 1997;18(6):356–364. 9208295 10.1177/107110079701800609

[18] DeVries JG , Taefi E , Bussewitz BW , Hyer CF , Lee TH . The fifth metatarsal base: anatomic evaluation regarding fracture mechanism and treatment algorithms. J Foot Ankle Surg. 2015;54(1):94–98. 25441854 10.1053/j.jfas.2014.08.019

[19] Eils E , Streyl M , Linnenbecker S , Thorwesten L , Völker K , Rosenbaum D . Characteristic plantar pressure distribution patterns during soccer‐specific movements. Am J Sports Med. 2004;32(1):140–145. 14754737 10.1177/0363546503258932

[20] Ercikti N , Apaydin N , Kocabiyik N , Yazar F . Insertional characteristics of the peroneus tertius tendon: revisiting the anatomy of an underestimated muscle. J Foot Ankle Surg. 2016;55(4):709–713. 26860045 10.1053/j.jfas.2016.01.018

[21] Fitch K , Blackwell J , Gilmour W . Operation for non‐union of stress fracture of the tarsal navicular. J Bone Joint Surg Br. 1989;71(1):105–110. 2644288 10.1302/0301-620X.71B1.2644288

[22] Gheewala R , Arain A , Rosenbaum AJ . Tarsal navicular fractures. Treasure Island, FL: StatPearls Publishing; 2025. 31194378

[23] Golano P , Fariñas O , Sáenz I . The anatomy of the navicular and periarticular structures. Foot Ankle Clin. 2004;9(1):1–23. 15062212 10.1016/S1083-7515(03)00155-4

[24] Gomes MR , Pinto AP , Fabián AA , Gomes TJM , Navarro A , Oliva XM . Insertional anatomy of peroneal brevis and longus tendon—a cadaveric study. Foot Ankle Surg. 2019;25(5):636–639. 30321932 10.1016/j.fas.2018.07.005

[25] Hasselman CT , Vogt MT , Stone KL , Cauley JA , Conti SF . Foot and ankle fractures in elderly white women. Incidence and risk factors. J Bone Joint Surg. 2003;85(5):820–824. 12728031 10.2106/00004623-200305000-00008

[26] Herterich V , Hofmann L , Böcker W , Polzer H , Baumbach SF . Acute, isolated fractures of the metatarsal bones: an epidemiologic study. Arch Orthop Trauma Surg. 2023;143(4):1939–1945. 35235028 10.1007/s00402-022-04396-3 PMC10030529

[27] Hoenig T , Eissele J , Strahl A , Popp KL , Stürznickel J , Ackerman KE , et al. Return to sport following low‐risk and high‐risk bone stress injuries: a systematic review and meta‐analysis. Br J Sports Med. 2023;57(7):427–432. 36720584 10.1136/bjsports-2022-106328

[28] Hong CC , Pearce CJ , Ballal MS , Calder JDF . Management of sports injuries of the foot and ankle: an update. Bone Joint J. 2016;98–B(10):1299–1311. 10.1302/0301-620X.98B10.37896 27694582

[29] Hosseini A , Dijk PV , Breuking S , Vopat B , Guss D , Johnson AH , et al. The peroneus brevis and plantar fascia insertions are related to proximal fifth metatarsal fractures. Foot Ankle Orthop. 2017;2(3).

[30] Imre N , Kocabiyik N , Sanal HT , Uysal M , Ozan H , Yazar F . The peroneus brevis tendon at its insertion site on fifth metatarsal bone. Foot Ankle Surg. 2016;22(1):41–45. 26869499 10.1016/j.fas.2015.04.009

[31] Joshi SD , Joshi SS , Athavale SA . Morphology of peroneus tertius muscle. Clin Anat. 2006;19(7):611–614. 16317742 10.1002/ca.20243

[32] Kane JM , Sandrowski K , Saffel H , Albanese A , Raikin SM , Pedowitz DI . The epidemiology of fifth metatarsal fracture. Foot Ankle Spec. 2015;8(5):354–359. 25666689 10.1177/1938640015569768

[33] Kaneko F , Edama M , Ikezu M , Matsuzawa K , Hirabayashi R , Kageyama I . Anatomic characteristics of tissues attached to the fifth metatarsal bone. Orthop J Sports Med. 2020;8(9):2325967120947725. 32995346 10.1177/2325967120947725 PMC7503013

[34] Karauda P , Paulsen F , Polguj M , Diogo R , Olewnik Ł . Morphological variability of the fibularis tertius tendon in human foetuses. Folia Morphol. 2022;81(2):451–457. 10.5603/FM.a2021.0039 33899207

[35] Keaveny TM , Wachtel EF , Ford CM , Hayes WC . Differences between the tensile and compressive strengths of bovine tibial trabecular bone depend on modulus. J Biomech. 1994;27(9):1137–1146. 7929463 10.1016/0021-9290(94)90054-x

[36] Kelikian AS . Sarrafian's anatomy of the foot and ankle: descriptive, topographic, functional. Philadelphia: Wolters Kluwer Health; Lippincott Williams & Wilkins; 2011.

[37] Khan M , Madden K , Burrus MT , Rogowski JP , Stotts J , Samani MJ , et al. Epidemiology and impact on performance of lower extremity stress injuries in professional basketball players. Sports Health. 2018;10(2):169–174. 29106811 10.1177/1941738117738988 PMC5857731

[38] Kiter E , Günal z , Karatosun V , Korman E . The relationship between the tibialis posterior tendon and the accessory navicular. Ann Anat Anat Anz. 2000;182(1):65–68. 10.1016/s0940-9602(00)80130-2 10668560

[39] Koç T , Bobuş Örs A , Kurtoglu Olgunus Z , Ozdemir AA . Evaluation of the relationship of navicular tuberosity and tibialis posterior tendon with medial longitudinal arch. Ann Anat. 2024;260:152663. 10.1016/j.aanat.2025.152663 40245997

[40] van Langelaan EJ . A kinematical analysis of the tarsal joints. An X‐ray photogrammetric study. Acta Orthop Scand Suppl. 1983;204:1–269. 6582753

[41] Lawrence SJ , Botte MJ . Jones' fractures and related fractures of the proximal fifth metatarsal. Foot Ankle. 1993;14(6):358–365. 8406253 10.1177/107110079301400610

[42] Manners‐Smith T . A study of the navicular in the human and anthropoid foot. J Anat Physiol. 1907;41(Pt 4):255–279. 17232739 PMC1289124

[43] Marshall D , MacFarlane RJ , Molloy A , Mason L . A review of the management and outcomes of tarsal navicular fracture. Foot Ankle Surg. 2020;26(5):480–486. 31229349 10.1016/j.fas.2019.05.020

[44] McInnis KC , Ramey LN . High‐risk stress fractures: diagnosis and management. PM R. 2016;8(3 Suppl):113–124. 10.1016/j.pmrj.2015.09.019 26972260

[45] McKeon KE , Johnson JE , McCormick JJ , Klein SE . The intraosseous and extraosseous vascular supply of the fifth metatarsal: implications for fifth metatarsal osteotomy. Foot Ankle Int. 2013;34(1):117–123. 23386771 10.1177/1071100712460227

[46] McKeon KE , McCormick JJ , Johnson JE , Klein SE . Intraosseous and extraosseous arterial anatomy of the adult navicular. Foot Ankle Int. 2012;33(10):857–861. 23050710 10.3113/FAI.2012.0857

[47] Mehta S , Zheng E , Heyworth BE , Rizzone K , Halstead M , Brown N , et al. Tarsal navicular bone stress injuries: a multicenter case series investigating clinical presentation, diagnostic approach, treatment, and return to sport in adolescent athletes. Am J Sports Med. 2023;51(8):2161–2168. 37265102 10.1177/03635465231170399

[48] Mengiardi B , Zanetti M , Schöttle PB , Vienne P , Bode B , Hodler J , et al. Spring ligament complex: MR imaging‐anatomic correlation and findings in asymptomatic subjects. Radiology. 2005;237(1):242–249. 16118154 10.1148/radiol.2371041065

[49] Morris PM , Francois AG , Marcus RE , Farrow LD . The effect of peroneus brevis tendon anatomy on the stability of fractures at the fifth metatarsal base. Foot Ankle Int. 2015;36(5):579–584. 25550452 10.1177/1071100714565177

[50] Niu W , Tang T , Zhang M , Jiang C , Fan Y . An in vitro and finite element study of load redistribution in the midfoot. Sci China Life Sci. 2014;57(12):1191–1196. 25249199 10.1007/s11427-014-4731-1

[51] Olewnik Ł . A proposal for a new classification for the tendon of insertion of tibialis posterior. Clin Anat. 2019;32(4):557–565. 30758860 10.1002/ca.23350

[52] Olewnik Ł . Fibularis tertius: anatomical study and review of the literature. Clin Anat. 2019;32(8):1082–1093. 31408221 10.1002/ca.23449

[53] Olewnik Ł , Wysiadecki G , Polguj M , Topol M . Anatomic study suggests that the morphology of the plantaris tendon may be related to Achilles tendonitis. Surg Radiol Anat. 2017;39(1):69–75. 27155667 10.1007/s00276-016-1682-1 PMC5309290

[54] Olson B , Wakeland W , Olson J , Zoghi S , Hubbard JK . A unique bilateral anatomical variation of the extensor digitorum longus and fibularis tertius muscles. Surg Radiol Anat. 2025;47:78. 39948201 10.1007/s00276-025-03591-2

[55] Omar H , Saini V , Wadhwa V , Liu G , Chhabra A . Spring ligament complex: illustrated normal anatomy and spectrum of pathologies on 3T MR imaging. Eur J Radiol. 2016;85(11):2133–2143. 27776669 10.1016/j.ejrad.2016.09.023

[56] Orendurff MS , Rohr ES , Segal AD , Medley JW , Green JR , Kadel NJ . Biomechanical analysis of stresses to the fifth metatarsal bone during sports maneuvers: Implications for fifth metatarsal fractures. Phys Sportsmed. 2009;37(2):87–92. 20048514 10.3810/psm.2009.06.1714

[57] Park J‐H , Kim D , Kwon H‐W , Lee M , Choi Y‐J , Park K‐R , et al. A new anatomical classification for tibialis posterior tendon insertion and its clinical implications: a cadaveric study. Diagnostics. 2021;11(9):1619. 34573961 10.3390/diagnostics11091619 PMC8466387

[58] Pasapula C , Ali AMS , Kiliyanpilakkil B , Hardcastle A , Koundu M , Gharooni A‐A , et al. High incidence of spring ligament laxity in ankle fractures with complete deltoid ruptures and secondary first ray instability. Foot. 2021;46:101720. 33531204 10.1016/j.foot.2020.101720

[59] Patil V , Ebraheim NA , Frogameni A , Liu J . Morphometric dimensions of the calcaneonavicular (spring) ligament. Foot Ankle Int. 2007;28(8):927–932. 17697659 10.3113/FAI.2007.0927

[60] Pavlov H , Torg JS , Freiberger RH . Tarsal navicular stress fractures: radiographic evaluation. Radiology. 1983;148(3):641–645. 6224230 10.1148/radiology.148.3.6224230

[61] Peeters K , Schreuer J , Burg F , Behets C , Van Bouwel S , Dereymaeker G , et al. Alterated talar and navicular bone morphology is associated with pes planus deformity: a CT‐scan study. J Orthop Res. 2013;31(2):282–287. 22991335 10.1002/jor.22225

[62] Pośnik M , Węgiel A , Zielinska N , Ruzik K , Olewnik Ł , Triantafyllou G , et al. Morphological variability and clinical significance of the fibularis tertius muscle: an extensive literature review. J Clin Med. 2025;14(11):3991. 40507753 10.3390/jcm14113991 PMC12155681

[63] Prathapamchandra V , Ravichandran P , Shanmugasundaram J , Jayaraman A , Salem RS . Vascular foramina of navicular bone: a morphometric study. Anat Cell Biol. 2017;50(2):93. 28713611 10.5115/acb.2017.50.2.93 PMC5509905

[64] Qatu M , Borrelli G , Traynor C , Weistroffer J , Jastifer J . Anatomy of the intermetatarsal facets of the fourth and fifth metatarsals. Foot Ankle Orthop. 2021;6(1):2473011420975709. 35097421 10.1177/2473011420975709 PMC8702710

[65] Raheja S , Choudhry R , Singh P , Tuli A , Kumar H . Morphological description of combined variation of distal attachments of fibulares in a foot. Surg Radiol Anat. 2005;27(2):158–160. 15580345 10.1007/s00276-004-0290-7

[66] Rajaram N , Srinivasan S , Verma S . Human navicular bone: a morphometric and morphological evaluation. Surg Radiol Anat. 2023;46(1):71–79. 37968490 10.1007/s00276-023-03259-9

[67] Richli W , Rosenthal D . Avulsion fracture of the fifth metatarsal: experimental study of pathomechanics. Am J Roentgenol. 1984;143(4):889–891. 6332501 10.2214/ajr.143.4.889

[68] Sammarco VJ . The talonavicular and calcaneocuboid joints: anatomy, biomechanics, and clinical management of the transverse tarsal joint. Foot Ankle Clin. 2004;9(1):127–145. 15062218 10.1016/S1083-7515(03)00152-9

[69] Sawah A , Kasture S , Bond A , Fisher L , Fisher A , Philpott M , et al. Anatomical description of the spring ligament articular facet. Foot Ankle Orthop. 2024;9(3):24730114241270207. 39193450 10.1177/24730114241270207 PMC11348484

[70] Schwagten K , Gill J , Thorisdottir V . Epidemiology of dancers fracture. Foot Ankle Surg. 2021;27(6):677–680. 33229215 10.1016/j.fas.2020.09.001

[71] Scott‐Moncrieff A , Forster BB , Andrews G , Khan K . The adult tarsal navicular: why it matters. Can Assoc Radiol J. 2007;58(5):279–285. 18286903

[72] Semple R , Murley GS , Woodburn J , Turner DE . Tibialis posterior in health and disease: a review of structure and function with specific reference to electromyographic studies. J Foot Ankle Res. 2009;2:24. 19691828 10.1186/1757-1146-2-24 PMC2739849

[73] Seyidova N , Hirtler L , Windhager R , Schuh R , Willegger M . Peroneus brevis tendon in proximal 5th metatarsal fractures: anatomical considerations for safe hook plate placement. Injury. 2018;49(3):720–725. 29357996 10.1016/j.injury.2018.01.008

[74] Shakked RJ , Walters EE , O'Malley MJ . Tarsal navicular stress fractures. Curr Rev Musculoskelet Med. 2017;10(1):122–130. 28110392 10.1007/s12178-017-9392-9 PMC5344863

[75] Shereff MJ , Yang QM , Kummer FJ , Frey CC , Greenidge N . Vascular anatomy of the fifth metatarsal. Foot Ankle. 1991;11(6):350–353. 1894227 10.1177/107110079101100602

[76] Smith CF . Anatomy, function, and pathophysiology of the posterior tibial tendon. Clin Podiatr Med Surg. 1999;16(3):399–406. 10470504

[77] Smith JW , Arnoczky SP , Hersh A . The intraosseous blood supply of the fifth metatarsal: implications for proximal fracture healing. Foot Ankle. 1992;13(3):143–152. 1601342 10.1177/107110079201300306

[78] Spang RC , Haber DB , Beaulieu‐Jones BR , Stupay KL , Sanchez G , Sanchez A , et al. Jones fractures identified at the national football league scouting combine: assessment of prognostic factors, computed tomography findings, and initial career performance. Orthop J Sports Med. 2018;6(8):2325967118790740. 30182027 10.1177/2325967118790740 PMC6113739

[79] Subramanian S , Chinnadurai CM , Latiff AA . Anatomical variations of peroneus tertius and its clinical implications: case report with systematic literature review. Surg Radiol Anat. 2023;45(11):1525–1533. 37742309 10.1007/s00276-023-03244-2

[80] Swanton E , Fisher L , Fisher A , Molloy A , Mason L . An anatomic study of the naviculocuneiform ligament and its possible role maintaining the medial longitudinal arch. Foot Ankle Int. 2019;40(3):352–355. 30466312 10.1177/1071100718811638

[81] Szaro P , Ghali Gataa K , Ciszek B . Anatomical variants of the medioplantar oblique ligament and inferoplantar longitudinal ligament: an MRI study. Surg Radiol Anat. 2022;44(2):279–288. 34800154 10.1007/s00276-021-02860-0 PMC8831290

[82] Taniguchi A , Tanaka Y , Takakura Y , Kadono K , Maeda M , Yamamoto H . Anatomy of the spring ligament. J Bone Joint Surg Am. 2003;85(11):2174–2178. 14630849 10.2106/00004623-200311000-00018

[83] Theodorou DJ , Theodorou SJ , Kakitsubata Y , Botte MJ , Resnick D . Fractures of proximal portion of fifth metatarsal bone: anatomic and imaging evidence of a pathogenesis of avulsion of the plantar aponeurosis and the short peroneal muscle tendon. Radiology. 2003;226(3):857–865. 12616022 10.1148/radiol.2263020284

[84] Torg JS , Balduini FC , Zelko RR , Pavlov H , Peff TC , Das M . Fractures of the base of the fifth metatarsal distal to the tuberosity. Classification and guidelines for non‐surgical and surgical management. J Bone Joint Surg Am. 1984;66(2):209–214. 6693447

[85] Torg JS , Pavlov H , Cooley LH , Bryant MH , Arnoczky SP , Bergfeld J , et al. Stress fractures of the tarsal navicular. A retrospective review of twenty‐one cases. J Bone Joint Surg Am. 1982;64(5):700–712. 7085695

[86] Tuthill HL , Finkelstein ER , Sanchez AM , Clifford PD , Subhawong TK , Jose J . Imaging of tarsal navicular disorders: a pictorial review. Foot Ankle Spec. 2014;7(3):210–224. 10.1177/1938640014528042 24686907

[87] Uchiyama I , Edama M , Yokota H , Hirabayashi R , Sekine C , Maruyama S , et al. Anatomical study of sites and surface area of the attachment region of tibial posterior tendon attachment. Int J Environ Res Public Health. 2022;19(24):16510. 36554392 10.3390/ijerph192416510 PMC9779476

[88] Vertullo CJ , Glisson RR , Nunley JA . Torsional strains in the proximal fifth metatarsal: implications for Jones and stress fracture management. Foot Ankle Int. 2004;25(9):650–656. 15563388 10.1177/107110070402500910

[89] Vieira A , Monteiro A , Nacur F , Coutinho R , Direito T , Torres D . Prevalence and topography of the peroneus tertius muscle: a study of human cadavers. J Morphol Sci. 2018;35(2):106–109.

[90] Whitmore I . Federative Committee on Anatomical Terminology. Terminologia anatomica: international anatomical terminology. Stuttgart: Thieme; 1998.

[91] Willegger M , Benca E , Hirtler L , Moser L , Zandieh S , Windhager R , et al. Peroneus brevis as source of instability in Jones fracture fixation. Int Orthop. 2020;44(7):1409–1416. 32372110 10.1007/s00264-020-04581-2 PMC7306048

[92] Willegger M , Seyidova N , Schuh R , Windhager R , Hirtler L . The tibialis posterior tendon footprint: an anatomical dissection study. J Foot Ankle Res. 2020;13(1):25. 32430082 10.1186/s13047-020-00392-1 PMC7236122

[93] Wright AA , Taylor JB , Ford KR , Siska L , Smoliga JM . Risk factors associated with lower extremity stress fractures in runners: a systematic review with meta‐analysis. Br J Sports Med. 2015;49(23):1517–1523. 26582192 10.1136/bjsports-2015-094828

[94] Wright RW , Fischer DA , Shively RA , Heidt RS , Nuber GW . Refracture of proximal fifth metatarsal (Jones) fracture after intramedullary screw fixation in athletes. Am J Sports Med. 2000;28(5):732–736. 11032233 10.1177/03635465000280051901

[95] Yammine K , Erić M . The fibularis (peroneus) tertius muscle in humans: a meta‐analysis of anatomical studies with clinical and evolutionary implications. BioMed Res Int. 2017;2017:6021707. 28596965 10.1155/2017/6021707 PMC5449999

[96] Yang H , Chen T , Xue F , Li J , Li Y . A case of musculi peronaeus tertius anatomic variation. Surg Radiol Anat. 2022;44(3):491–494. 35254492 10.1007/s00276-022-02899-7

[97] Yildiz S , Yalcin B . An unique variation of the peroneus tertius muscle. Surg Radiol Anat. 2012;34(7):661–663. 22223362 10.1007/s00276-011-0929-0

